# Field comparison of BG-Pro configurations with CDC light traps and evaluation of the BG-Counter 2 trap for automated mosquito surveillance in urban and rural Thailand

**DOI:** 10.1186/s13071-026-07503-0

**Published:** 2026-06-13

**Authors:** Vanutsarin Anakerit, Songpol Eiamsam-ang, Yudthana Samung, Anon Phayakkaphon, Sirasate Bantuchai, Martin Geier, Michael Weber, Myat Su Yin, Saranath Lawpoolsri, Peter Haddawy, Patchara Sriwichai

**Affiliations:** 1 Department of Medical Entomology, Faculty of Tropical Medicine, Mahidol University, Ratchathewi, Bangkok, Thailand; 2 Mahidol Vivax Research Unit, Faculty of Tropical Medicine, Mahidol University, Bangkok, Thailand; 3 Biogents AG, Regensburg, Germany; 4 Department of Tropical Hygiene, Faculty of Tropical Medicine, Mahidol University, Bangkok, Thailand; 5 Faculty of ICT, Mahidol University, 999 Phuttamonthon 4 Road, Salaya, Nakhon Pathom Thailand; 6 Bremen Spatial Cognition Center, University of Bremen, Bremen, Germany

**Keywords:** Surveillance system, Mosquito trap, Mosquito-borne diseases, Trapping efficacy, Automatic counting

## Abstract

**Background:**

Entomological surveillance programmes need traps that perform reliably across vector taxa and ecological settings, and automated tools that reduce labour without compromising data quality. We evaluated BG-Pro trap configurations and the BG-Counter 2 against the standard CDC light trap (CDC-LT) across contrasting urban and rural ecologies in Thailand.

**Methods:**

We compared CDC light traps (CDC-LT) baited with CO₂, BG-Pro traps (BG-Lure + CO₂), and inverted BG-Pro traps (BG-Lure + CO₂) using a 3 × 3 Latin square design over 270 trap-days across two urban sites (Bangkok and Nakhon Pathom; Nov 2024–Mar 2025) and one rural malaria-endemic site (Tha Song Yang, Tak; Jul — Oct 2024). Trap performance was analysed using generalized linear mixed models (GLMMs) to estimate incidence rate ratios (IRRs). The BG-Counter 2 accuracy was assessed over 60 trap-days by comparing automated with manual counts.

**Results:**

A total of 54,054 mosquitoes representing six genera were collected, dominated by *Culex* (77.6%). Across sites, BG-Pro produced the highest overall catches (urban mean 946.04; rural mean 13.29 mosquitoes/trap-day) and outperformed CDC-LT in both urban (IRR = 6.11) and rural (IRR = 2.64) settings, while the inverted BG-Pro traps captured the fewest mosquitoes. Genus-specific patterns were consistent across ecological settings: BG-Pro achieved the highest mean capture rates for *Aedes* (urban 8.3; rural 1.4 mosquitoes/trap-day) and *Culex* (urban 936.3; rural 8.3 mosquitoes/trap-day), indicating stronger performance for culicines relative to CDC-LT. In the rural site, overall captures of *Anopheles minimus* were very low across all traps, while CDC-LT sampled a broader range of anopheline taxa. The BG-Counter 2 median accuracy was high in urban sites (89.0%) but low in the rural site (20.6%), with the sensor overcounting mosquitoes on the majority of trap-days in both settings.

**Conclusions:**

No single trap optimally samples all vector groups across ecological settings. BG-Pro (BG-Lure + CO₂) showed promising utility for operational culicine surveillance in urban Thailand, whereas CDC-LT remains preferable for anopheline monitoring in rural malaria-risk settings. The BG-Counter 2 can support near-real-time trend monitoring in low-background urban environments but requires site-specific calibration before rural deployment. These findings support a context-specific surveillance strategy that combines complementary traps rather than relying on a single platform.

**Graphical Abstract:**

**Figure:**
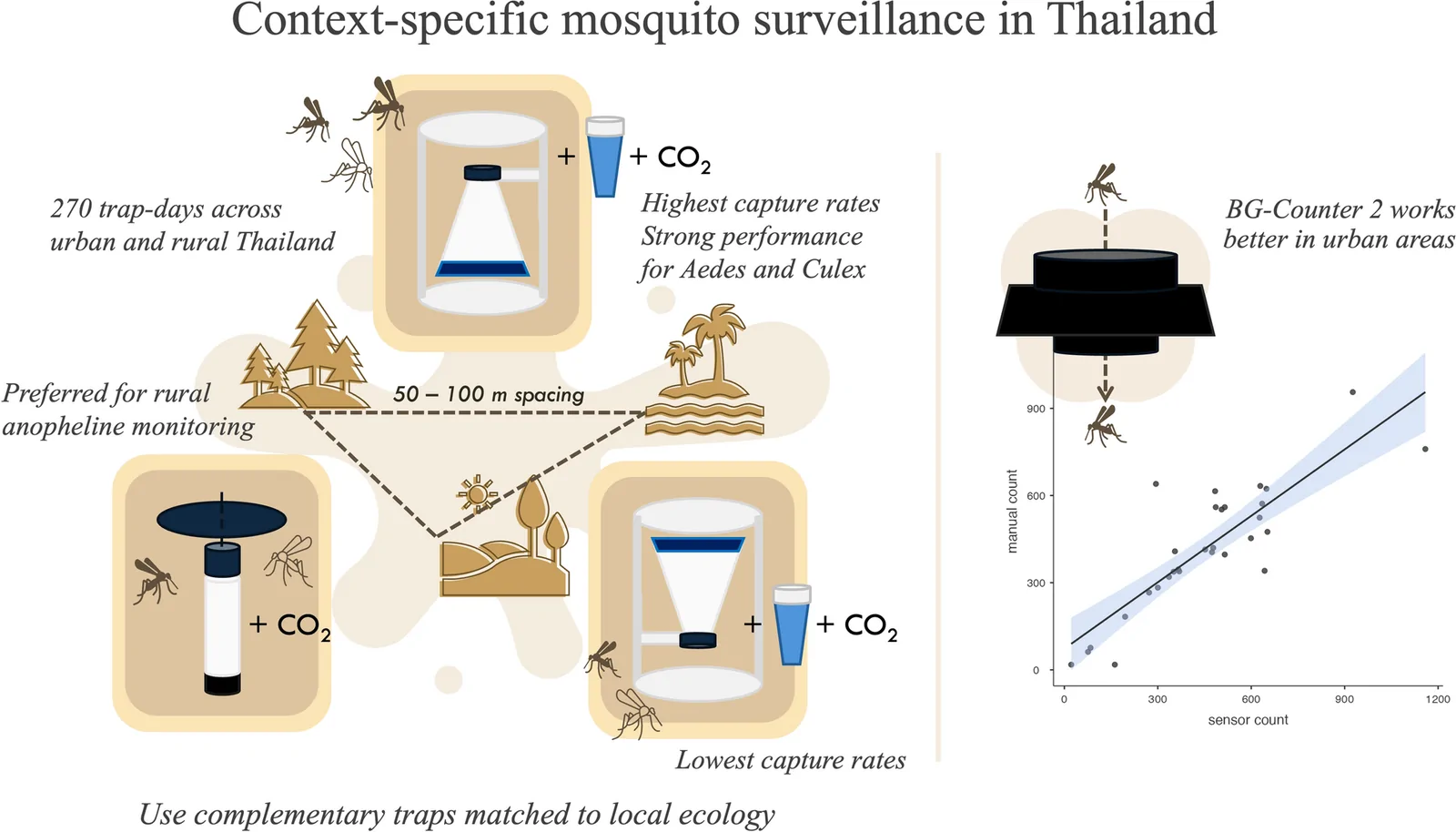

**Supplementary Information:**

The online version contains supplementary material available at 10.1186/s13071-026-07503-0.

## Background

Vector-borne diseases continue to pose a major global public health challenge. Mosquitoes transmit pathogens that are responsible for dengue and other arboviral diseases as well as malaria. Nearly half of the world’s population is at risk, and the health and economic burdens remain substantial [1–3]. Rapid urbanization, inadequate waste management, and changing land use have expanded peri-domestic breeding habitats, particularly in tropical settings, while climate variability and warming temperatures are shifting mosquito distributions and accelerating pathogen development within vectors [4]. These factors are reshaping mosquito abundance and species composition across urban–rural gradients and highlight the need for surveillance systems that can reliably detect changes in vector populations and guide timely, cost-effective control.

Thailand faces concurrent mosquito-borne disease threats in both rural and urban settings. Malaria transmission remains concentrated in border provinces such as Tak, Mae Hong Son, and Kanchanaburi, where forested habitats and population movement sustain risk and *Plasmodium vivax* accounts for a large proportion of infections [5]. At the same time, dengue and other arboviruses persist in urban and peri-urban areas, requiring surveillance that can support targeted intervention. Ongoing challenges—including ecological change, climate variability, insecticide resistance, and vector re-emergence in areas previously considered low-risk—can delay outbreak detection and hinder timely response.

Entomological surveillance underpins early warning systems and evaluation of vector control. The human landing catch (HLC) directly measures human–mosquito contact and remains a reference method for sampling host-seeking mosquitoes [6], but it is unsuitable for routine large-scale surveillance because of ethical concerns and exposure risk. The CDC miniature light trap (CDC-LT) is therefore widely used as an operational alternative for malaria vector monitoring [7]. Although cost-effective and easy to deploy, CDC-LT performance varies by species and setting, and the light source can attract non-target insects, complicate processing and potentially affecting downstream analyses. In Thailand, Sriwichai et al. [8] evaluated CDC-LT with and without dry ice in indoor and outdoor environments along the Thai Myanmar border, collecting 1,832 *Anopheles* over 1,600 trap-nights (1.15 mosquitoes/night). Ponlawat and colleagues further reported that CDC-LT baited with BG-Lure plus CO₂ outperformed the BG-Sentinel trap for malaria vector surveillance, capturing 973 female *Anopheles* over 54 trap-nights [9].

Monitoring mosquito populations in field settings is complicated by variation in vector behaviour across species, season, and habitat, and surveillance systems must balance sensitivity with practicality and cost. Operational programmes also face major constraints, including labour-intensive trapping routines, time-consuming taxonomic identification, and shortages of trained entomologists. These limitations have increased interest in newer trap platforms and automated tools that can improve capture efficiency and reduce manual workload. However, in Thailand there is limited independent evidence comparing newer modular trap configurations—including inverted (downward-intake) designs—against CDC-LT across contrasting urban and rural settings. In addition, field validation of automated counting devices under high background insect activity remains scarce, leaving uncertainty about where these tools provide operational advantages and how reliably they estimate mosquito abundance.

The BG-Pro (BGP) is a modular mosquito trap system that supports multiple configurations and can be integrated with the BG-Counter 2—an automated device designed to remotely classify and count insects, record environmental parameters, and transmit time-stamped data in near real time [10]. Field evaluations suggest that the BGP can perform as well as or better than several commercial traps (e.g., BG-Sentinel, CDC, EVS, BG-Mosquitaire) for capturing *Aedes*, *Culex*, and *Culiseta* mosquitoes [11]. BG-based systems have also been adapted for anopheline surveillance; for example, Batista et al. demonstrated successful collection of *Anopheles gambiae* in semi-field conditions [12]. However, the BGP platform is relatively costly and operationally complex, and independent evidence is still needed to define where it offers meaningful advantages over simpler traps in routine surveillance.

Automated counting devices may increase surveillance throughput and enable high-resolution monitoring of temporal trends, but field-based validation of the BG-Counter 2 remains limited and reported accuracy varies widely. Published studies indicate that accuracy depends strongly on mosquito abundance and environmental conditions, with frequent over- and undercounting unless calibrated [10, 13]. Misclassification may increase in environments with high non-target insect activity, where insects overlap with mosquitoes in wingbeat frequency and size. The BG-Counter 2 may therefore be useful for tracking relative trends in mosquito activity, but robust ground-truth validation is required before it can be used to estimate absolute mosquito abundance across ecological settings.

This study aimed to compare BG-Pro configurations (standard and inverted) with CDC-LT across urban and rural settings in Thailand and validated the BG-Counter 2 automated counts against manual identification. The findings provide practical guidance on the integrated use of the BGP trap and the BG-Counter 2, contributing to updating evidence on trap performance across Thai ecological settings and vector communities. Additionally, this study highlights both advantages and limitations of these tools, informing entomologists and vector control programmes in their decision-making for vector surveillance strategies.

## Methods

### Study site

Trap comparison experiments were carried out in one rural and two urban locations in Thailand. Study sites were classified based on population density and land cover/land use. Tha Song Yang District, Tak Province, is a malaria-endemic rural area with a population density of approximately 50 people per km^2^ and a high proportion of forest cover (64%) relative to built-up area (1%). Trapping was conducted during the rainy and early cool seasons (July–October 2024). The two urban sites were in Bangkok and Nakhon Pathom Provinces, where built-up land cover predominated (81.5%) and tree cover is low (1.6%); trapping was conducted during the cool and hot seasons (November 2024–March 2025). Temperature and relative humidity during trapping were obtained from the BG-Counter 2 system and summarized by setting (rural mean 29 °C, range 16–41 °C; 66% RH; urban mean 34 °C, range 28–42 °C; 55% RH). Land cover metrics were derived from Copernicus Sentinel-2 data (Copernicus Data Space Ecosystem) (https://dataspace.copernicus.eu).

### Study design

A 3 × 3 Latin square design was used to compare three trap configurations: the CDC miniature light trap (CDC-LT), the BG-Pro trap (BGP), and the inverted BG-Pro (IBG). Trapping was conducted across two ecological settings: an urban setting represented by trap locations in Bangkok and Nakhon Pathom, and a rural malaria-endemic setting in Tha Song Yang, Tak. For analysis, Bangkok and Nakhon Pathom were treated as one urban setting. A total of 270 trap-days were completed, comprising 135 trap-days in the urban setting and 135 trap-days in the rural setting. In each setting, three trap types were operated simultaneously at three fixed trap positions over 3 consecutive days and rotated among positions every 24 h. This 3-day Latin square cycle was replicated 15 times per setting, resulting in 135 trap-days per setting (3 traps × 3 days × 15 cycles) and 270 trap-days overall. Trap positions were spaced approximately 100 m apart where feasible, with a minimum distance of 50 m when field conditions were constrained, to reduce interference between attractant plumes. All traps were deployed in comparable peri-domestic outdoor environments within each setting, and collections were retrieved at the same time each day. Only replicates that completed the full 3-day Latin square cycle were included in the comparative analyses.

### Trap configurations and operation

#### CDC miniature light trap (CDC-LT)

The CDC Miniature Light Trap Model 512 (CDC-LT, BioQuip, USA) was suspended approximately 1.5 m above the ground. Mosquitoes were attracted by an incandescent bulb positioned above a battery-powered fan (6-V). No LED or UV light source was used in the CDC-LT configuration in this study, instead a battery-powered fan (6-V) was positioned above it, with the battery placed on the ground and connected by wire. This incandescent-light configuration was selected to reflect the conventional CDC-LT setup used in routine vector surveillance programmes and to allow comparison with previous field studies in Thailand. Mosquitoes were collected in a long white catch bag attached to the collection cup. In this study, each CDC-LT was hung from house eaves or tree branches approximately 1.5 m above the ground (100 m from other traps). Traps were baited with 1 kg of dry ice as CO_2_ source.

#### BG-Pro trap (BGP)

The BG-Pro mosquito trap (Biogents AG, Regensburg, Germany) was placed on a metal BG-Trap station approximately 40 cm above the ground. The trap was baited with 1 kg of dry ice as a CO₂ source and a BG-Lure. BG-Sweetscent was used in the urban sites to target culicine mosquitoes, whereas BG-Mb5 was used in the rural site to target anopheline mosquitoes. Importantly, the optional BG-Pro LED light ring was not used in this study; therefore, no LED or UV light source was applied to the BG-Pro traps. This configuration was selected to evaluate the performance of odour-baited BG-Pro traps and to reduce non-target insect attraction that may occur when light sources are used. The cone-shaped fabric trap included a three-blade fan operating at 5–6 V and was powered by a 10,000 mAh rechargeable power bank. Mosquitoes were collected in a catch bag through a funnel above the fan. Each BG-Pro trap was set up approximately 100 m from other traps.

#### Inverted BG-Pro (IBG)

The IBG used the same BG-Pro components, CO₂, and lure as the standard BGP but was deployed in an inverted orientation. The optional LED light ring was also not used in the inverted configuration. This configuration was included to evaluate whether trap orientation influences capture efficiency and species composition under field conditions.

#### BG-Counter 2 integration

The BG-Counter 2 (Biogents AG, Regensburg, Germany) was mounted on the upper bracket of the BG-Pro trap according to the manufacturer’s instructions. The device classifies and counts flying insects based on wingbeat frequency and signal characteristics and transmits time-stamped data wirelessly to a cloud server for remote monitoring.

#### BG-Counter 2 accuracy evaluation

The BG-Counter 2 performance was evaluated under field conditions, in both urban and rural settings over 60 trap-days. For each trap-day, automated counts were extracted as the total number of [mosquito-classified/all flying-insect] events recorded during the trapping period and compared with manual counts from the corresponding catch bag. The BG-Counter 2 counting accuracy was calculated for each trap-day as: min (manual, sensor) / max (manual, sensor) × 100, where manual is the hand-count of mosquitoes recovered from the corresponding catch bag, and sensor is the automated sensor count. This symmetric metric reports the proportion of counts in agreement regardless of direction. Accuracy is summarized using the median, as the distribution was strongly right skewed in the rural setting. To characterize the direction of counting error, trap-days were classified as overcounting (sensor > manual), undercounting (sensor < manual), or exact (sensor = manual), and the proportion of trap-days in each category is reported separately for rural and urban settings.

### Mosquito processing and identification

Mosquitoes were collected from the traps using a collection bag during each daily sampling period. Genus and species were identified using standard morphological keys [14–18]. Sex was recorded for all specimens, and female physiological status was classified (unfed/empty, blood-fed, and gravid) by trained entomology staff based on morphological identification where applicable.

### Sample preservation and transportation

All mosquito specimens were preserved in a sealed container on dry ice immediately after collection and transported from the field site to the Department of Medical Entomology at the Faculty of Tropical Medicine, Mahidol University. Upon arrival at the laboratory, specimens were stored in the freezer at –20 °C to prevent degradation.

### Statistical analysis

Mosquito abundance was summarized as the number of mosquitoes per trap-day by site, trap type, genus/species, and sex (Additional file 1: Table S1). Statistical analyses compared the catch performance of all three trap types: BG-Pro (BGP), IBG, and CDC-LT. Catch rate and species diversity analyses used manual mosquito counts throughout; BG-Counter 2 automated sensor counts were assessed separately as described below. Trap performance (mosquito counts per trap-day) was analysed using generalized linear mixed models (GLMMs) fitted with the glmmTMB package in R. Three candidate distributions were compared by AIC: Poisson, Negative Binomial type 2 (NB2), and Zero-Inflated Negative Binomial (ZINB). The NB2 model was selected as best-fitting (AIC = 2,356.5; θ = 0.689). Model assumptions were verified using DHARMa simulation-based diagnostics, including formal tests for overdispersion and zero-inflation (Table 1). CDC-LT was used as the reference category for estimating incidence rate ratios (IRRs). Trap type was included as a fixed effect, and the Latin square structure was accounted for by including replicate (3-day cycle) as a random intercept (1 | exp), and day nested within experiment as a further random intercept (1 | exp:day). Trap position was not included as a separate random effect, as positional effects are controlled by the Latin square rotation. Setting (urban and rural) was included as a fixed effect, and an interaction term (trap type × setting) was used to test whether relative trap performance differed by setting. Incidence rate ratios (IRR) and 95% confidence intervals were calculated by exponentiating model coefficients, with pairwise trap comparisons conducted using emmeans with Tukey adjustment.

**Table 1:** Model selection summary

Model	Distribution	AIC	ΔAIC	θ	SD (exp)	SD (exp:day)
M1	Poisson	21,912.56	19,556.04	–	0.867	0.560
M2	Negative binomial (NB2)	2,356.52	0.00	0.689	0.699	< 0.001
M3	ZINB	2,357.22	0.71	0.757	0.708	< 0.001

*AIC* Akaike Information Criterion; *ΔAIC* difference from best model; *θ* NB2 dispersion parameter; *SD* standard deviation of random intercept

Mosquito diversity was assessed using Shannon’s diversity index. Shannon H was computed independently for each trap-day (29 species, 270 trap-days; 90 values per trap type) using the vegan package in R. Differences among trap types were evaluated using Kruskal–Wallis tests followed by pairwise comparisons using Dunn's test with Bonferroni correction, applied overall and separately within each location. Pielou's evenness index (J = H / ln S, where S is the number of species with count > 0) was also computed per trap-day for trap-days with more than one species present. Only complete 3-day Latin square replicates were included in the analysis.

The accuracy of the BG-Counter 2 was calculated as described above, and median accuracy was compared between urban and rural settings using a Wilcoxon rank-sum test. Pearson’s and Spearman’s correlation coefficients were used to evaluate the relationship between sensor counts and manual counts. All statistical analyses were performed using R (version 2024.4.0) using the glmmTMB, DHARMa, emmeans, vegan, and dunn.test packages.

## Results

### Mosquito abundance and species composition

Across all trap configurations and study sites, 54,054 mosquitoes representing six genera were collected. Species richness differed by setting, with 14 species identified in the urban sites (Bangkok and Nakhon Pathom) and 18 species in the rural site (Tak) (Additional file 1: Table S1). *Culex* dominated collections (77.6% of all specimens), driven primarily by *Culex. quinquefasciatus* (*n* = 41,951). Other common *Culex* species included *Cx. vishnui* (*n* = 5,031), *Cx. gelidus* (*n* = 4,403), and *Cx. tritaeniorhynchus* (*n* = 1,239), which together account for 97.5% of *Culex* captures. *Aedes* were less frequently collected (*Aedes aegypti n* = 421, *Ae. albopictus n* = 121). Among anophelines, *Anopheles paraliae* (*n* = 235) and *An. peditaeniatus* (*n* = 131) were the most common, with smaller numbers of *An. minimus* (*n* = 25) and *An. maculatus* (*n* = 4). *Mansonia* species (*Mansonia annulifera n* = 59; *Ma. uniformis n* = 43; *Ma. indiana n* = 42), along with low numbers of *Tripteroides* spp. and *Armigeres* spp., were also recorded. Overall, culicine mosquitoes predominated in both settings, with anopheline species occurring mainly at the rural site.

### Overall trap performance

Across 270 trap-days, the mean daily catches varied among trap configurations (Table 2). The BG-Pro (BGP) captured the highest number of mosquitoes per trap-day in both settings, followed by CDC-LT, while the inverted BG-Pro (IBG) consistently yielded the lowest catches. In urban sites, the BGP collected a mean of 946.0 ± 935.6 mosquitoes/trap-day (median 694), whereas catches were substantially lower at the rural site (13.3 ± 21.4 mosquitoes/trap-day, median = 5) (Table 2).

**Table 2:** Average mosquito captures per trap-day in urban and rural settings

Trap configurations	Urban Mean ± SD (median)	Rural Mean ± SD (median)
BGP	946.04 ± 935.55 (Median = 694)^a^	13.29 ± 21.43 (Median = 5)^a^
CDC-LT	173.76 ± 285.13 (Median = 64)^b^	4.18 ± 5.25 (Median = 2)^b^
IBG	62.89 ± 102.66 (Median = 14)^c^	1.04 ± 1.71 (Median = 0)^c^
Kruskal–Wallis (*χ*^*2*^)	65.4	31.7

*CDC-LT*; CDC light trap, *BGP*; BG-Pro mosquito trap, *IBG*; Invert BG-Pro mosquito trap. ^a,b,c^Difference lowercase letters indicate statistically significant differences among trap within each setting (Dunn's test, P < 0.05)

The Poisson model was rejected due to severe overdispersion (AIC = 21,912.6; ΔAIC = 19,556.0). The NB2 model was selected as best-fitting (AIC = 2,356.5; θ = 0.689). The ZINB model offered no improvement (ΔAIC = 0.71), indicating the 19.3% zero counts were consistent with the NB2 distribution. DHARMa diagnostics showed no residual overdispersion (ratio = 0.244, p = 0.172) or zero-inflation (ratio = 1.022, p = 0.942). Between-experiment clustering, the experiment-level random effect was substantial (random effect SD = 0.699), while day-within-experiment variance was negligible (SD < 0.001) validating the chosen specification*.* Model selection results are summarized in Table 1.

Generalized linear mixed models (GLMMs) confirmed these differences (Table 3): the BGP collected more mosquitoes than CDC-LT in both urban (IRR = 6.11, 95% CI: 3.25 – 11.47, *P* < 0.001) and rural (IRR = 2.64, 95% CI: 1.37 – 5.08, *P* = *0.002*) settings, whereas IBG collected fewer mosquitoes than CDC-LT (urban IRR = 0.37, 95% CI: 0.20 – 0.69, P < 0.001; rural IRR = 0.24, 95% CI: 0.11 – 0.51, P < 0.001). Trap type and setting both had significant main effects, and the trap type × setting interaction was not significant, indicating a consistent ranking of traps across settings (BGP > CDC-LT > IBG). Higher mosquito collections were observed in lure-equipped traps (BGP) across study setting. However, because different lure formulations were used between urban and rural settings, the independent contributions of attractant formulation and ecological setting to trap performance could not be fully distinguished.

**Table 3:** Incidence rate ratios (IRR) comparison of three trap configurations across urban and rural setting

Setting	Trap configurations	*IRR*	95%CI	*z*	*P*-value
Urban	CDC-LT + CO_2_ (Reference)	1.00	–	–	–
	BGP + BG-Lure^a^ + CO_2_	6.11	3.25 – 11.47	6.03	< 0.001^***^
	IBG + BG-Lure^a^ + CO_2_	0.37	0.20 – 0.69	−3.74	< 0.001^***^
Rural	CDC-LT + CO_2_ (Reference)	1.00	–	–	–
	BGP + BG-Lure^a^ + CO_2_	2.64	1.37 – 5.08	3.99	0.0015^**^
	IBG + BG-Lure^a^ + CO_2_	0.24	0.11 – 0.51	−4.40	< 0.001^***^

*CDC-LT*, CDC light trap; *BGP*, BG-Pro trap; *IBG*, inverted BG-Pro; *IRR*, incidence rate ratio; *CI*, confidence interval. ^a^BG-Lure formulation differed by setting (urban: BG-Sweetscent; rural: BG-Mb5). IRRs were estimated using generalized linear mixed models (GLMMs) comparing BGP and IBG trap configurations against CDC-LT (reference) in urban and rural settings. Models used negative binomial distribution with nested random effects (day within experimental replicate), significant difference when *P < 0.05, **P < 0.01, ***P < *0.001*

### Species-specific trap efficiency

Of all mosquitoes collected, 87.7% of specimens were female and 99.0% of females were unfed, consistent with host-seeking collections. Species-specific analyses showed that the BGP (BG-Lure + CO₂) increased capture rates for several dominant culicine taxa relative to CDC-LT (Table 4). In urban sites, the BGP captured substantially more *Ae. aegypti* (IRR = 13.58, 95% CI: 7.49 – 24.61, P < 0.001) and *Cx. quinquefasciatus* (9.89, 95% CI: 5.94 – 16.45, P < 0.001) than CDC-LT, and differences for *Cx. tritaeniorhynchus* were not statistically significant (IRR = 1.33, 95% CI: 0.42 – 4.24, P = 0.63). These effects were consistent with mean daily catches per the BGP trap (*Ae. aegypti* ~ 8; *Cx. quinquefasciatus* ~ 808; *Cx. tritaeniorhynchus* ~ 14 mosquitoes per trap-day; Additional file 2: Table S2). In the rural site, the BGP similarly increased *Cx. quinquefasciatus* (IRR = 7.14, 95% CI: 3.38 – 15.05, P < 0.001), whereas differences for *Aedes* were not statistically significant (Table 4). In contrast, the BGP did not capture significantly more mosquitoes than CDC-LT for anophelines. Although the BGP collected slightly more *An. minimus* (mean 0.3 ± 0.8; Additional file 2: Table S2), the low sample size precluded formal statistical comparison. CDC-LT sampled a broader range of anopheline taxa in the rural site, supporting its continued utility for malaria vector surveillance. The IBG generally produced the lowest catches across taxa (Table 4).

**Table 4:** Incidence rate ratios (IRRs) for major vector species captured by three trap configurations in urban and rural settings

	Species	Trap comparison^a^	IRR (95%CI)	*P*-value
Urban				
	*Aedes aegypti*	BGP vs CDC-LT	13.58 (7.49–24.61)	< 0.001^***^
		IBG vs CDC-LT	1.15 (0.57–2.34)	0.69
	*Aedes albopictus*	BGP vs CDC-LT	2.51 (0.65–9.79)	0.18
		IBG vs CDC-LT	0.22 (0.03–1.50)	0.12
	*Culex quinquefasciatus*	BGP vs CDC-LT	9.89 (5.94–16.45)	< 0.001^***^
		IBG vs CDC-LT	0.42 (0.25–0.72)	0.002^**^
	*Culex tritaeniorhynchus*	BGP vs CDC-LT	1.33 (0.42–4.24)	0.63
		IBG vs CDC-LT	0.15 (0.05–0.48)	0.001^**^
	*Culex vishnui*	BGP vs CDC-LT	1.26 (0.61–2.59)	0.53
		IBG vs CDC-LT	0.45 (0.20–1.04)	0.06
	*Mansonia annulifera*	BGP vs CDC-LT	0.73 (0.43–1.23)	0.24
		IBG vs CDC-LT	0.06 (0.01–0.25)	< 0.001^***^
	*Mansonia indiana*	BGP vs CDC-LT	0.40 (0.15–1.07)	0.07
		IBG vs CDC-LT	0.07 (0.01–0.33)	< 0.001^***^
	*Mansonia uniformis*	BGP vs CDC-LT	0.05 (0.01–0.47)	0.009^**^
		IBG vs CDC-LT	–	–
Rural				
	*Aedes aegypti*	BGP vs CDC-LT	4.78 (0.86–26.43)	0.073
		IBG vs CDC-LT	0.47 (0.04–5.96)	0.56
	*Aedes albopictus*	BGP vs CDC-LT	2.25 (0.93–5.45)	0.074
		IBG vs CDC-LT	0.07 (0.01–0.35)	0.0015^**^
	*Anopheles minimus*	BGP vs CDC-LT	1.45 (0.43–4.92)	0.55
		IBG vs CDC-LT	0.67 (0.16–2.76)	0.57
	*Culex quinquefasciatus*	BGP vs CDC-LT	7.14 (3.38–15.05)	< 0.001^***^
		IBG vs CDC-LT	0.62 (0.25–1.53)	0.30

^a^*CDC-LT*; CDC light trap (CO_2_-bait), *BGP*; BG-Pro mosquito trap (BG-lure^b^ + CO_2_-bait), *IBG*; Invert BG-Pro mosquito trap (BG-lure^b^ + CO_2_-bait), *IRR:* Incident Rate Ratio*, CI*: Confidence Interval, corresponding *P*-value based on GLMMs with negative binomial distribution with nested random effects (day within experimental replicate). "–" indicates model did not converge, significant difference when *P* less than 0.05^*^, 0.01^**^, 0.001^***^. ^b^BG-Lure; BG-Sweetscent (urban), BG-Mb5 (rural)

### Mosquito diversity indices

Mosquito community diversity differed between settings and among trap types. Shannon diversity index (H) was computed per trap-day (29 species, 270 trap-days; 90 values per trap type). Overall, Shannon H differed significantly among trap types (Kruskal–Wallis χ^2^ = 31.09, df = 2, p < 0.0001). The IBG showed markedly lower species diversity (mean H = 0.227 ± 0.367; median = 0.000) than the BGP (mean H = 0.501 ± 0.498; median = 0.353) or LT (mean H = 0.503 ± 0.508; median = 0.338). Dunn post hoc tests confirmed the IBG differed significantly from both BGP and LT (p < 0.0001 each), while BGP and LT did not differ (p = 0.690). This effect was driven entirely by rural trap-days (χ^2^ = 30.62, p < 0.0001); no significant difference was observed in urban settings (χ^2^ = 2.42, p = 0.298). Evenness (Pielou's J) was computed per trap-day for trap-days with more than one species present (BGP: n = 76; IBG: n = 33; LT: n = 62). Overall, J did not differ significantly among trap types (Kruskal–Wallis χ^2^ = 5.79, df = 2, p = 0.055). Within urban settings, J differed significantly (χ^2^ = 21.57, df = 2, p < 0.0001), while no significant difference was observed in rural settings (χ^2^ = 2.95, df = 2, p = 0.229).

In the rural study area, *Anopheles* captures were limited across all trap types. The CDC-LT captured the greatest *Anopheles* species richness (7 species; mean 0.53 individuals per trap-day), compared with the IBG (4 species; mean 0.18 per trap-day) and BG-Pro (2 species; mean 0.38 per trap-day). In urban settings, species richness was comparable across traps (BG-Pro and CDC-LT: 3 species each; IBG: 2 species), although the CDC-LT yielded substantially higher total counts (mean 5.47 per trap-day), driven primarily by *An. paraliae* (n = 234). Total *An. minimus* captures in the rural study area were n = 25 across all trap types combined (BG-Pro: 12, IBG: 5, CDC-LT: 8). Given this small sample, formal statistical comparison of *An. minimus* catches among trap types was not performed.

### BG-Counter 2 accuracy and background interference

The BG-Counter 2 performance was evaluated over 30 trap-days per environment (urban and rural) by comparing automated counts with manual processing of catch bags every 24 h. The sensor classified flying insects into three categories (mosquitoes, small insects, large insects) based on signal features including wingbeat frequency and size. Daily accuracy was calculated following Day et al. [10], applying daily counting accuracy to measure the relative agreement between two count methods. The BG-Counter 2 median accuracy was 89.0% (range 11.1 – 99.4%, n = 30) in the urban setting and 20.6% (range 0–100%, n = 30) in the rural setting; accuracy differed significantly between settings (Wilcoxon rank-sum W = 58.5, p < 0.0001). In the urban setting, the sensor overcounted on 73.3% (22/30) of trap-days and undercounted on 26.7% (8/30), with a median absolute error of 46.0 mosquitoes. In the rural setting, the sensor overcounted on 93.3% (28/30) of trap-days and undercounted on 3.3% (1/30), with a median absolute error of 20.5 mosquitoes. The predominance of overcounting in both settings suggests a systematic positive bias in the BG-Counter 2 sensor counts relative to manual enumeration.

### Association between sensor and manual mosquito counts

**Fig. 1:**
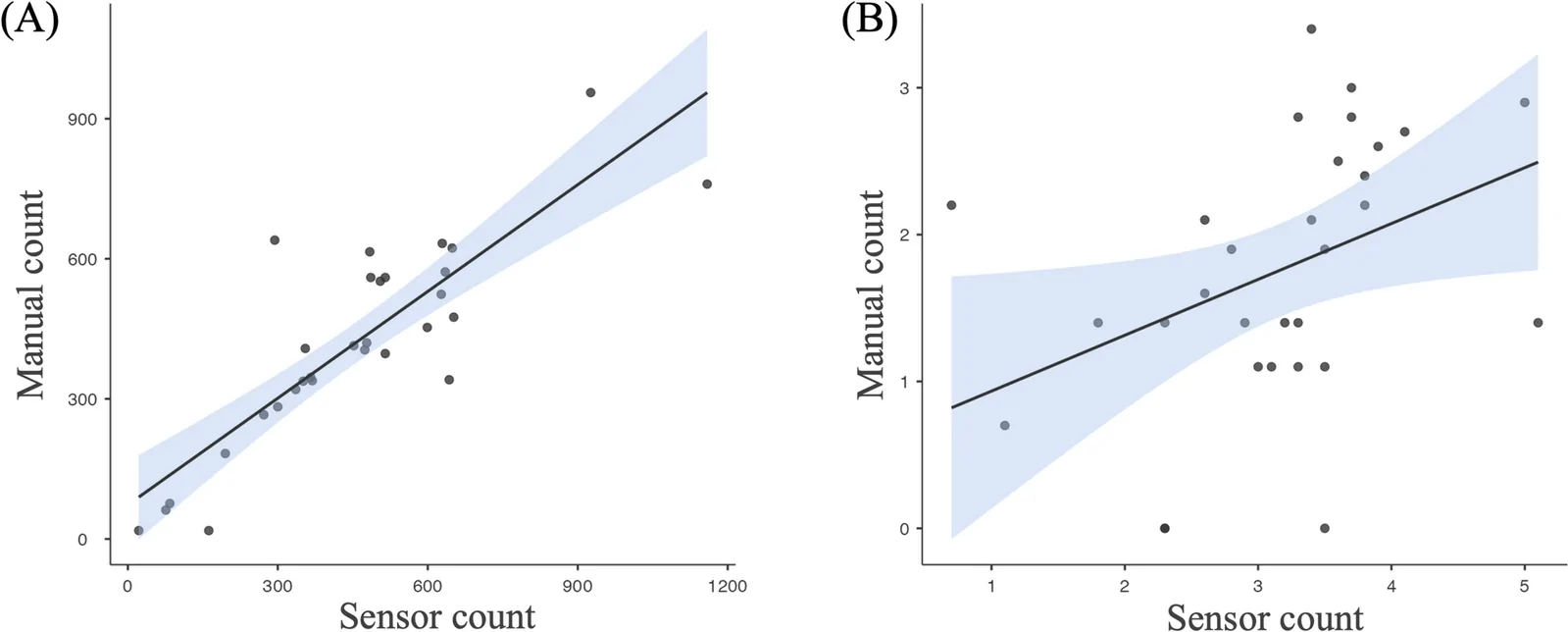
Scatterplots showing the relationship between BG-Counter 2 automated count and manual mosquito count in urban (**A**) and rural (**B**) settings

Although absolute accuracy was lower in the rural site, automated counts remained positively associated with manual counts in both settings (Fig. 1). In urban sites, sensor counts were strongly correlated with manual counts (Pearson *r* = 0.850, *df* = 28, *P* < 0.001). In the rural site, where count distributions were non-normal, the association was moderate but significant (Spearman *ρ* = 0.521, *df* = 28, *P* = 0.003). These results suggest that the BG-Counter 2 captured relative temporal variation in mosquito abundance, although precision was lower under rural conditions with higher background insect activity.

### Mosquito activity patterns and background interference

**Fig. 2:**
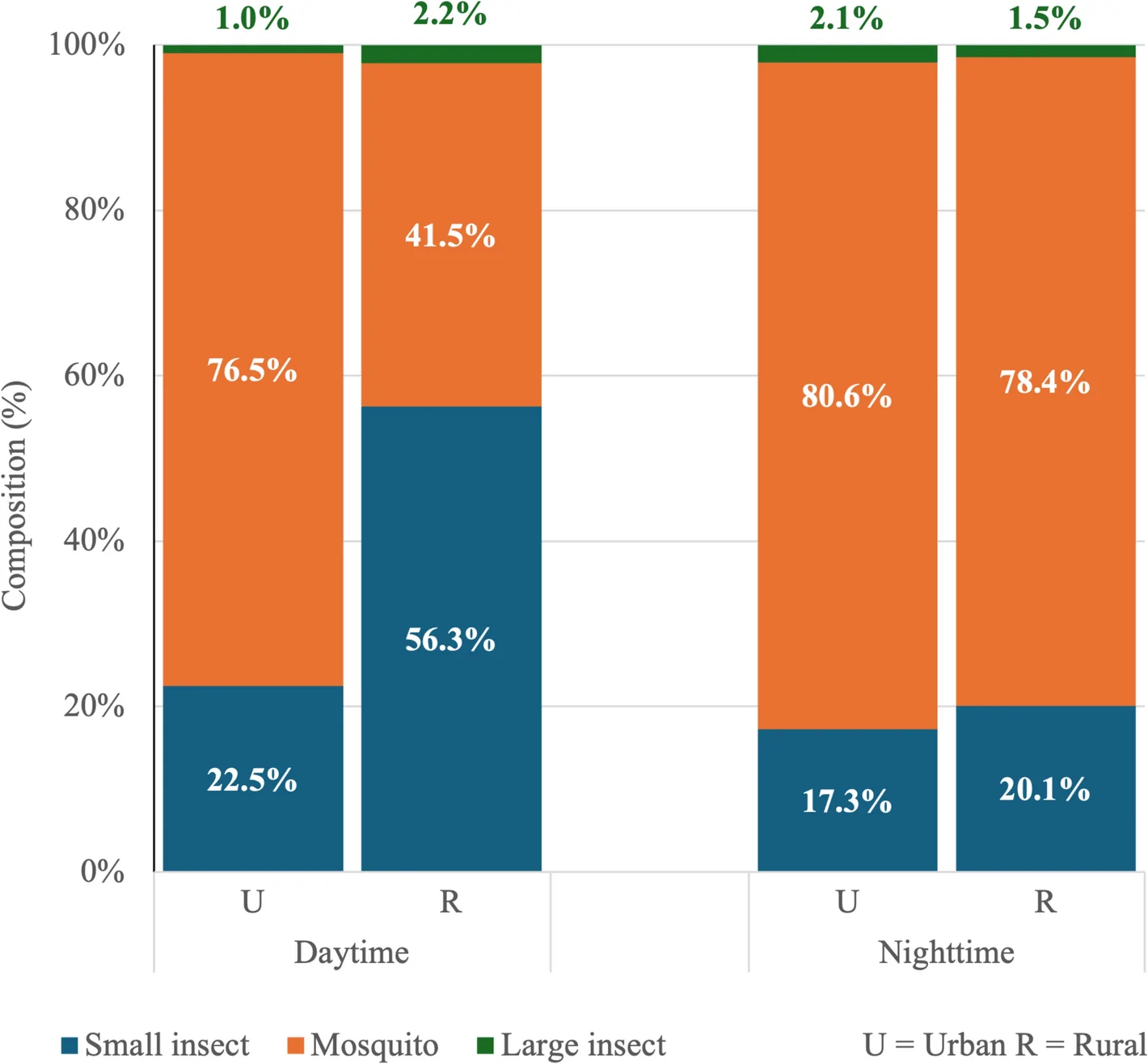
Proportion of BG-Counter2 detections classified as small insects, mosquitoes, and large insects during daytime and nighttime in urban and rural settings

Time-stamped the BG-Counter 2 detections were aggregated to compare nighttime (18:00–06:00) and daytime (06:00–18:00) activity over the 24-h collection period (Fig. 2). Mosquito detections were higher at night in both settings, with a secondary daytime increase observed in urban sites. Background insect activity was higher in the rural site and overlapped with mosquito detections. During rural daytime periods, “small insects” comprised 56.3% of detections compared with 41.5% classified as mosquitoes and 2.2% as large insects (Fig. 2), likely contributing to increased misclassification and reduced the BG-Counter 2 accuracy. In addition, some large-bodied mosquitoes (e.g., *Armigeres subalbatus*) were occasionally classified as “large insects”, indicating that non-target insects can affect automated classification.

## Discussion

Mosquito surveillance is essential for evidence-based vector control, yet trap performance varies substantially across taxa and ecological settings. In this study, we conducted a field comparison of three trap configurations (BG-Pro, inverted BG-Pro, and CDC light trap) across urban and rural environments in Thailand and evaluated the operational reliability of the BG-Counter 2 automated counting device. BG-Pro consistently increased overall mosquito collections relative to CDC-LT (urban IRR = 6.11, 95% CI: 3.25 – 11.47, P < 0.001; rural IRR = 2.64, 95% CI: 1.37 – 5.08, P = 0.0015), whereas BG-Pro offered no clear advantage for anopheline capture in the malaria-endemic rural site, where CDC-LT captured greater *Anopheles* species richness in rural settings (7 species; mean 0.53 individuals per trap-day) compared with the BG-Pro (2 species; mean 0.38 per trap-day) and the IBG (4 species; mean 0.18 per trap-day). However, urban and rural trapping were conducted in different seasons (rural: July–October 2024; urban: November 2024–March 2025), and the trap x location interaction cannot fully separate the ecological setting from seasonal effects. In addition, the higher mosquito collections observed in lure-equipped traps suggest that attractant formulations may have contributed to increased trapping efficiency. Because lure type and ecological setting were fully confounded in the present study, their independent effects could not be clearly separated. Therefore, differences in BG-Pro performance between urban and rural settings should be interpreted cautiously, as they may reflect both ecological variation and lure-related effects. Furthermore, urban and rural sites were sampled during different seasonal periods, which may have additionally influenced mosquito abundance and community composition. Nevertheless, these findings support a fit-for-purpose approach to mosquito surveillance rather than reliance on a single “universal” trap across all ecological settings.

### BG-Pro strengthens culicine surveillance, especially for dominant urban taxa

BG-Pro performance for culicine mosquitoes was consistent with prior evaluations of BG-based traps showing high capture efficiency for *Aedes* and *Culex* species. In our urban sites, BG-Pro increased captures of key culicine vectors, including *Ae. aegypti* (IRR = 13.58, *P* < 0.001) and *Cx. quinquefasciatus* (IRR = 9.89, *P* < 0.001) compared with CDC-LT (Table 4), supporting the view that CO₂ combined with human-odour lures can strongly enhance attraction to host-seeking culicines [9, 21]. Such gains are operationally important in urban settings, where rapid detection of increasing *Ae. aegypti* populations can improve the timing and targeting of dengue prevention efforts. It should be noted, however, that urban and rural trapping were conducted in different seasons (urban: November 2024-March 2025 cool/hot; rural: July–October 2024 rainy/cool), and seasonal differences in mosquito abundance and community composition may have contributed to the observed urban–rural contrast alongside differences in land use and vector community structure.

The IBG configuration was included as an exploratory assessment based on previous studies suggesting that trap orientation and airflow direction may influence mosquito attraction and capture efficiency under field conditions [12, 19]. Therefore, this modified deployment was evaluated to investigate its potential utility under the ecological settings examined in the present study. However, its consistently poor performance suggests that inversion may disrupt trap entry dynamics and odour plume presentation, reducing attraction or entry.

### CDC-LT remains operationally valuable for anopheline surveillance

Despite strong culicine performance, the BG-Pro did not demonstrate a clear advantage over CDC-LT for anopheline capture in the rural site. The CDC-LT captured greater *Anopheles* species richness in the rural site (7 species; mean 0.53 individuals per trap-day) compared with the BG-Pro (2 species; mean 0.38 per trap-day) and the IBG (4 species; mean 0.18 per trap-day). Total *An. minimus* captures were limited to n = 25 across all trap types and trap-nights (BG-Pro: 12, IBG: 5, CDC-LT: 8), precluding formal statistical comparison among traps. This low anopheline capture likely reflects the sensitivity of anopheline responses to local vector behaviour, microhabitat, deployment height, airflow characteristics, and lure specificity [19–21]. In Thailand’s malaria-endemic contexts [22], the CDC-LT remains a practical tool for routine malaria vector surveillance, consistent with earlier Thai field work [9], though future studies with greater sampling effort are needed to formally evaluate trap performance for *An. minimus*.

### Implications: integrated “two-trap” strategy may maximize epidemiological coverage

The divergent performance profiles observed here underscore the importance of matching surveillance tools to the target vector group and ecology [23, 24]. In Thailand and similar setting, a practical strategy may involve using BG-Pro for culicine surveillance in urban and peri-urban areas and CDC-LT for anopheline monitoring in rural malaria-risk areas. Operational constraints should also be weighed, including trap cost, power and maintenance needs, and CO₂/lure logistics [2, 25]. Importantly, the BG-Lure formulation differed between urban and rural deployments (BG-Sweetscent vs BG-Mb5) (Table 4). This difference may partially confound setting-level comparisons and should be considered when interpreting urban–rural differences (Tables 5 and 6)

**Table 5:** Shannon diversity index (H) and Pielou evenness index (J) of mosquito communities by trap configuration

Trap	Shannon H Mean	Shannon H SD	Shannon H Median	Pielou J Mean (n)	Kruskal–Wallis test (*p-value*)
BGP	0.501	0.498	0.353	0.477 (n = 76)	
CDC-LT	0.503	0.508	0.338	0.596 (n = 62)	
IBG	0.227	0.367	0.000	0.584 (n = 33)	
Shannon H KW	–	–	–	–	χ^2^ = 31.09, p < 0.0001
Pielou J KW	–	–	–	–	χ^2^ = 5.79, p = 0.055

*CDC-LT*; CDC light trap (CO_2_-bait), *BGP*; BG-Pro mosquito trap (BG-lure + CO_2_-bait), *IBG*; Invert BG-Pro mosquito trap (BG-lure + CO_2_-bait). Only complete 3-day Latin square replicates were included. P-values from Kruskal–Wallis test

**Table 6:** BG-Counter 2 counting accuracy compared with manual mosquito identification in urban and rural settings

Setting	n	Median accuracy (%)	Range (%)	Overcount n (%)	Undercount n (%)	Median abs error	Wilcoxon W (p)
Urban	30	89.0	11.1–99.4	22 (73.3%)	8 (26.7%)	46.0	W = 58.5, p < 0.0001
Rural	30	20.6	0–100	28 (93.3%)	1 (3.3%)	20.5	

Accuracy was non-normally distributed (Shapiro–Wilk test); median and range are reported, and the Wilcoxon rank-sum test was used for urban–rural comparison. MAE = median absolute error (mosquitoes per trap-day). Overcount: automated count > manual count; undercount: automated count < manual count

### BG-Counter 2: suitable for urban trend monitoring but limited for absolute counts in biodiverse rural settings

Automated counting can increase surveillance throughput and enable near real-time monitoring, but performance may be constrained by background non-target insect activity. In our study, the BG-Counter 2 produced substantially higher accuracy or higher relative agreement (%) in urban sites, and sensor counts were strongly associated with manual counts, supporting its use for tracking temporal trends where mosquito detections dominate. Accuracy declined sharply in the rural environment, consistent with previous reports of misclassification and context-dependent performance under field conditions [10, 13]. Our findings indicate that the BG-Counter 2 may be best used for trend detection in urban settings in biodiverse rural environment, however, it likely requires calibration, algorithm refinement, and periodic ground-truthing to support reliable inference on absolute abundance. Despite capturing fewer individuals and lower species richness (Shannon H), The IBG trap-days showed comparable or higher evenness (Pielou's J), particularly in urban settings (Kruskal–Wallis χ^2^ = 21.57, p < 0.0001). This suggests the IBG's low catches are not biased toward a single dominant species but reflect a general reduction in capture efficiency across all species present. In contrast, the BGP catches were heavily dominated by *Culex quinquefasciatus*, resulting in lower evenness despite higher total abundance and species richness. These patterns indicate that BGP and LT provide a more representative picture of the local mosquito community overall, while the IBG consistently under samples across species.

## Strengths and limitations

Key strengths include a large sample size (>54,000 mosquitoes), a robust Latin square design, and the inclusion of epidemiologically important taxa across contrasting ecological settings. Limitations include seasonal differences between sites, evaluation of only one attractant package per trap, potential confounding due to the use of different BG-Lure formulations used across settings, and the small number of *An. minimus* captured in the rural study area (n = 25 total across all trap types), which was insufficient to support a formal statistical comparison of trap performance for this malaria vector. In addition, the collections in rural and urban settings were conducted during different seasonal periods, which may have influenced mosquito abundance and community composition. Consequently, the independent contributions of ecology, seasonality, and lure formulation to observed differences in trap performance could not be fully disentangled. Another consideration is the difference in trap placement height between trap types, which may have influenced mosquito capture patterns by sampling different vertical flight strata or species-specific flight behaviour. Future work should standardize lure packages across environments, test deployment heights/configurations optimized for anophelines, quantify non-target insect loads to improve automated classification under rural conditions, and increase sampling effort at sites and seasons known to be higher *An. minimus* activity to achieve adequate power for malaria vector trap comparisons.

## Conclusions

This study provides field-based evidence on trap performance and automated counting under urban and rural conditions in Thailand. BG-Pro baited with CO₂ and BG-Lure substantially improved culicine surveillance—particularly *Ae. aegypti* and *Cx. quinquefasciatus*—in urban environments, whereas CDC-LT remained an operationally useful tool for anopheline monitoring in the rural malaria-endemic site. The BG-Counter 2 showed greater reliability in urban settings but limited agreement between methods in rural environments with high non-target insect interference, showing that automated surveillance requires ecological validation and calibration. However, these findings should be interpreted with consideration of seasonal differences between collection periods and the lure formulations used in each setting. No single trap is optimal across vector groups and settings. Integrated surveillance systems that combine complementary traps with calibrated automation are likely to provide the most robust evidence for vector-control decision-making in heterogeneous environments.

## Supplementary Information

Additional file 1. Table S1

Additional file 2. Table S2

## Data Availability

Data supporting the main conclusions of this study are included in the manuscript.

## Abbreviations

BGP: BG-Pro mosquito trap
CDC-LT: CDC light trap
IBG: Inverted BG-Pro
IRR: Incidence rate ratio
CI: Confidence interval
HLC: Human landing catch
GLMMs: Generalized linear mixed models
