# Supplementary material for: Field comparison of BG-Pro configurations with CDC light traps and evaluation of the BG-Counter 2 trap for automated mosquito surveillance in urban and rural Thailand
Source: Parasit Vectors. 2026 Jun 13;19:343. doi: 10.1186/s13071-026-07503-0 (PMC13491874; doi:10.1186/s13071-026-07503-0)
Supplement: Supplementary file 1 [file 13071_2026_7503_MOESM1_ESM.docx]

Additional file 1: Table S1. Mosquitoes collection across three trap configurations in urban and rural setting.

| Setting | Species | Trap method | | | | | | | | | | | |
| --- | --- | --- | --- | --- | --- | --- | --- | --- | --- | --- | --- | --- | --- |
|  |  | CDC-LT + CO2 | | | | BGP + BG-Lure*^a^* + CO_2_ | | | | IBG + BG-Lure*^a^* + CO_2_ | | | |
|  |  | Total (Mean) | | Male | Female | Total (Mean) | | Male | Female | Total (Mean) | | Male | Female |
| Urban | Number of trap days | 45 |  |  |  | 45 |  |  |  | 45 |  |  |  |
|  | *Aedes aegypti* | 28 | (0.6) | 7 | 21 | 349 | (7.8) | 202 | 147 | 31 | (0.7) | 24 | 7 |
|  | *Aedes albopictus* | 9 | (0.2) | 3 | 6 | 24 | (0.5) | 11 | 13 | 2 | (0) | 1 | 1 |
|  | *Anopheles paraliae* | 234 | (5.2) | 1 | 233 | 1 | (0) | 0 | 1 | 0 | (0) | 0 | 0 |
|  | *Anopheles peditaeniatus* | 7 | (0.2) | 0 | 7 | 18 | (0.4) | 0 | 18 | 105 | (2.3) | 0 | 105 |
|  | *Anopheles vagus* | 5 | (0.1) | 1 | 4 | 3 | (0.1) | 1 | 2 | 2 | (0) | 1 | 1 |
|  | *Culex gelidus* | 1419 | (31.5) | 24 | 1395 | 2579 | (57.3) | 30 | 2549 | 403 | (9) | 9 | 394 |
|  | *Culex pseudovishnui* | 45 | (1) | 0 | 45 | 0 | (0) | 0 | 0 | 1 | (0) | 0 | 1 |
|  | *Culex quinquefasciatus* | 3247 | (72.2) | 194 | 3053 | 36392 | (808.7) | 5463 | 30929 | 1938 | (43.1) | 444 | 1494 |
|  | *Culex tritaeniorhynchus* | 518 | (11.5) | 0 | 518 | 652 | (14.5) | 0 | 652 | 67 | (1.5) | 0 | 67 |
|  | *Culex vishnui* | 2199 | (48.9) | 3 | 2196 | 2509 | (55.8) | 6 | 2503 | 275 | (6.1) | 8 | 267 |
|  | *Culex whitmorei* | 1 | (0) | 0 | 1 | 0 | (0) | 0 | 0 | 0 | (0) | 0 | 0 |
|  | *Culex spp.* | 1 | (0) | 0 | 1 | 2 | (0) | 0 | 2 | 0 | (0) | 0 | 0 |
|  | *Mansonia annulifera* | 33 | (0.7) | 2 | 31 | 24 | (0.5) | 2 | 22 | 2 | (0) | 0 | 2 |
|  | *Mansonia indiana* | 30 | (0.7) | 6 | 24 | 10 | (0.2) | 0 | 10 | 2 | (0) | 0 | 2 |
|  | *Mansonia uniformis* | 41 | (0.9) | 18 | 23 | 2 | (0) | 0 | 2 | 0 | (0) | 0 | 0 |
|  | *Mansonia spp.* | 1 | (0) | 0 | 1 | 0 | (0) | 0 | 0 | 0 | (0) | 0 | 0 |
|  | *Tripteroides spp.* | 1 | (0) | 0 | 1 | 7 | (0.2) | 0 | 7 | 2 | (0) | 1 | 1 |
|  | Total No. | 7819 |  | 259 | 7560 | 42572 |  | 5715 | 36857 | 2830 |  | 488 | 2342 |
|  |  |  |  |  |  |  |  |  |  |  |  |  |  |
| Rural | Number of trap days | 45 |  |  |  | 45 |  |  |  | 45 |  |  |  |
|  | *Aedes aegypti* | 2 | (0) | 1 | 1 | 10 | (0.2) | 4 | 6 | 1 | (0) | 1 | 0 |
|  | *Aedes albopictus* | 30 | (0.7) | 2 | 28 | 54 | (1.2) | 7 | 47 | 2 | (0) | 1 | 1 |
|  | *Aedes poicilius* | 2 | (0) | 0 | 2 | 0 | (0) | 0 | 0 | 0 | (0) | 0 | 0 |
|  | *Aedes spp.* | 4 | (0.1) | 0 | 4 | 19 | (0.4) | 0 | 19 | 0 | (0) | 0 | 0 |
|  | *Anopheles annularis* | 1 | (0) | 0 | 1 | 0 | (0) | 0 | 0 | 1 | (0) | 0 | 1 |
|  | *Anopheles culicifacies* | 6 | (0.1) | 0 | 6 | 0 | (0) | 0 | 0 | 0 | (0) | 0 | 0 |
|  | *Anopheles dirus* | 1 | (0) | 0 | 1 | 0 | (0) | 0 | 0 | 0 | (0) | 0 | 0 |
|  | *Anopheles maculatus* | 4 | (0.1) | 1 | 3 | 0 | (0) | 0 | 0 | 0 | (0) | 0 | 0 |
|  | *Anopheles minimus* | 8 | (0.2) | 0 | 8 | 12 | (0.3) | 0 | 12 | 5 | (0.1) | 0 | 5 |
|  | *Anopheles peditaeniatus* | 1 | (0) | 0 | 1 | 0 | (0) | 0 | 0 | 0 | (0) | 0 | 0 |
|  | *Anopheles tessellatus* | 0 | (0) | 0 | 0 | 0 | (0) | 0 | 0 | 1 | (0) | 0 | 1 |
|  | *Anopheles spp.* | 3 | (0.1) | 0 | 3 | 5 | (0.1) | 1 | 4 | 1 | (0) | 0 | 1 |
|  | *Armigeres subalbatus* | 72 | (1.6) | 6 | 66 | 117 | (2.6) | 7 | 110 | 3 | (0.1) | 0 | 3 |
|  | *Culex gelidus* | 0 | (0) | 0 | 0 | 2 | (0) | 0 | 2 | 0 | (0) | 0 | 0 |
|  | *Culex quinquefasciatus* | 30 | (0.7) | 10 | 20 | 323 | (7.2) | 116 | 207 | 21 | (0.5) | 9 | 12 |
|  | *Culex tritaeniorhynchus* | 0 | (0) | 0 | 0 | 2 | (0) | 0 | 2 | 0 | (0) | 0 | 0 |
|  | *Culex vishnui* | 15 | (0.3) | 1 | 14 | 25 | (0.6) | 0 | 25 | 8 | (0.2) | 0 | 8 |
|  | *Culex fuscocephala* | 0 | (0) | 0 | 0 | 2 | (0) | 0 | 2 | 0 | (0) | 0 | 0 |
|  | *Culex whitmorei* | 0 | (0) | 0 | 0 | 0 | (0) | 0 | 0 | 1 | (0) | 0 | 1 |
|  | *Culex spp.* | 4 | (0.1) | 0 | 4 | 21 | (0.5) | 0 | 21 | 1 | (0) | 0 | 1 |
|  | *Mansonia annulata* | 0 | (0) | 0 | 0 | 1 | (0) | 0 | 1 | 1 | (0) | 0 | 1 |
|  | *Mansonia spp.* | 1 | (0) | 0 | 1 | 1 | (0) | 0 | 1 | 0 | (0) | 0 | 0 |
|  | *Tripteroides spp.* | 4 | (0.1) | 0 | 4 | 4 | (0.1) | 0 | 4 | 1 | (0) | 0 | 1 |
|  | Total No. | 188 |  | 21 | 167 | 598 |  | 135 | 463 | 47 |  | 11 | 36 |

*Abbreviations: CDC-LT; CDC light trap, BGP; BG-Pro mosquito trap, IBG; Invert BG-Pro mosquito trap, ^a^BG-Lure; BG-Sweetscent (urban), BG-Mb5 (rural).*
