# Supplementary material for: Field comparison of BG-Pro configurations with CDC light traps and evaluation of the BG-Counter 2 trap for automated mosquito surveillance in urban and rural Thailand
Source: Parasit Vectors. 2026 Jun 13;19:343. doi: 10.1186/s13071-026-07503-0 (PMC13491874; doi:10.1186/s13071-026-07503-0)
Supplement: Supplementary file 2 [file 13071_2026_7503_MOESM2_ESM.docx]

Additional file 2: Table S2. Average daily capture across three trap configurations in urban and rural setting.

| Trap | Setting | Species | N | Mean | Median | SD | Minimum | Maximum |
| --- | --- | --- | --- | --- | --- | --- | --- | --- |
| BGP | urban | *Aedes aegypti* | 45 | 7.7556 | 4 | 11.651 | 0 | 72 |
|  |  | *Aedes albopictus* | 45 | 0.5333 | 0 | 1.829 | 0 | 10 |
|  |  | *Anopheles paraliae* | 45 | 0.0222 | 0 | 0.149 | 0 | 1 |
|  |  | *Anopheles peditaeniatus* | 45 | 0.4000 | 0 | 2.683 | 0 | 18 |
|  |  | *Anopheles vagus* | 45 | 0.0667 | 0 | 0.330 | 0 | 2 |
|  |  | *Culex gelidus* | 45 | 57.3111 | 3 | 121.612 | 0 | 529 |
|  |  | *Culex quinquefasciatus* | 45 | 808.7111 | 319 | 1003.709 | 3 | 4210 |
|  |  | *Culex spp.* | 45 | 0.0444 | 0 | 0.298 | 0 | 2 |
|  |  | *Culex tritaeniorhynchus* | 45 | 14.4889 | 0 | 35.921 | 0 | 139 |
|  |  | *Culex vishnui* | 45 | 55.7556 | 2 | 130.275 | 0 | 513 |
|  |  | *Mansonia annulifera* | 45 | 0.5333 | 0 | 1.766 | 0 | 11 |
|  |  | *Mansonia indiana* | 45 | 0.2222 | 0 | 0.636 | 0 | 3 |
|  |  | *Mansonia uniformis* | 45 | 0.0444 | 0 | 0.208 | 0 | 1 |
|  | rural | *Aedes aegypti* | 45 | 0.2222 | 0 | 0.704 | 0 | 4 |
|  |  | *Aedes albopictus* | 45 | 1.2000 | 0 | 2.651 | 0 | 15 |
|  |  | *Culex gelidus* | 45 | 0.0444 | 0 | 0.298 | 0 | 2 |
|  |  | *Culex quinquefasciatus* | 45 | 7.1778 | 0 | 17.026 | 0 | 70 |
|  |  | *Culex spp.* | 45 | 0.4667 | 0 | 1.618 | 0 | 9 |
|  |  | *Culex tritaeniorhynchus* | 45 | 0.0444 | 0 | 0.298 | 0 | 2 |
|  |  | *Culex vishnui* | 45 | 0.5556 | 0 | 1.307 | 0 | 6 |
|  |  | *Mansonia spp.* | 45 | 0.0222 | 0 | 0.149 | 0 | 1 |
|  |  | *Tripteroides spp.* | 45 | 0.0889 | 0 | 0.596 | 0 | 4 |
|  |  | *Aedes spp.* | 45 | 0.4222 | 0 | 2.388 | 0 | 16 |
|  |  | *Anopheles minimus* | 45 | 0.2667 | 0 | 0.837 | 0 | 4 |
|  |  | *Anopheles spp.* | 45 | 0.1111 | 0 | 0.318 | 0 | 1 |
|  |  | *Armigeres subalbatus* | 45 | 2.6000 | 1 | 4.059 | 0 | 19 |
|  |  | *Culex fuscocephala* | 45 | 0.0444 | 0 | 0.208 | 0 | 1 |
|  |  | *Mansonia annulata* | 45 | 0.0222 | 0 | 0.149 | 0 | 1 |
| IBG | urban | *Aedes aegypti* | 45 | 0.6889 | 0 | 1.607 | 0 | 9 |
|  |  | *Aedes albopictus* | 45 | 0.0444 | 0 | 0.208 | 0 | 1 |
|  |  | *Anopheles peditaeniatus* | 45 | 2.3333 | 0 | 15.652 | 0 | 105 |
|  |  | *Anopheles vagus* | 45 | 0.0444 | 0 | 0.298 | 0 | 2 |
|  |  | *Culex gelidus* | 45 | 8.9556 | 0 | 30.199 | 0 | 171 |
|  |  | *Culex pseudovishnui* | 45 | 0.0222 | 0 | 0.149 | 0 | 1 |
|  |  | *Culex quinquefasciatus* | 45 | 43.0667 | 4 | 96.608 | 0 | 525 |
|  |  | *Culex tritaeniorhynchus* | 45 | 1.4889 | 0 | 4.536 | 0 | 27 |
|  |  | *Culex vishnui* | 45 | 6.1111 | 0 | 17.173 | 0 | 97 |
|  |  | *Mansonia annulifera* | 45 | 0.0444 | 0 | 0.208 | 0 | 1 |
|  |  | *Mansonia indiana* | 45 | 0.0444 | 0 | 0.208 | 0 | 1 |
|  |  | *Tripteroides spp.* | 45 | 0.0444 | 0 | 0.208 | 0 | 1 |
|  | rural | *Aedes aegypti* | 45 | 0.0222 | 0 | 0.149 | 0 | 1 |
|  |  | *Aedes albopictus* | 45 | 0.0444 | 0 | 0.298 | 0 | 2 |
|  |  | *Culex quinquefasciatus* | 45 | 0.4667 | 0 | 1.198 | 0 | 6 |
|  |  | *Culex spp.* | 45 | 0.0222 | 0 | 0.149 | 0 | 1 |
|  |  | *Culex vishnui* | 45 | 0.1778 | 0 | 0.442 | 0 | 2 |
|  |  | *Culex whitmorei* | 45 | 0.0222 | 0 | 0.149 | 0 | 1 |
|  |  | *Tripteroides spp.* | 45 | 0.0222 | 0 | 0.149 | 0 | 1 |
|  |  | *Anopheles annularis* | 45 | 0.0222 | 0 | 0.149 | 0 | 1 |
|  |  | *Anopheles minimus* | 45 | 0.1111 | 0 | 0.318 | 0 | 1 |
|  |  | *Anopheles spp.* | 45 | 0.0222 | 0 | 0.149 | 0 | 1 |
|  |  | *Anopheles tessellatus* | 45 | 0.0222 | 0 | 0.149 | 0 | 1 |
|  |  | *Armigeres subalbotus* | 45 | 0.0667 | 0 | 0.447 | 0 | 3 |
|  |  | *Mansonia annulata* | 45 | 0.0222 | 0 | 0.149 | 0 | 1 |
| LT | urban | *Aedes aegypti* | 45 | 0.6222 | 0 | 1.284 | 0 | 5 |
|  |  | *Aedes albopictus* | 45 | 0.2000 | 0 | 0.505 | 0 | 2 |
|  |  | *Anopheles paraliae* | 45 | 5.2000 | 0 | 32.111 | 0 | 215 |
|  |  | *Anopheles peditaeniatus* | 45 | 0.1556 | 0 | 0.673 | 0 | 4 |
|  |  | *Anopheles vagus* | 45 | 0.1111 | 0 | 0.611 | 0 | 4 |
|  |  | *Culex gelidus* | 45 | 31.5333 | 1 | 76.704 | 0 | 398 |
|  |  | *Culex pseudovishnui* | 45 | 1.0000 | 0 | 6.267 | 0 | 42 |
|  |  | *Culex quinquefasciatus* | 45 | 72.1556 | 23 | 113.492 | 0 | 528 |
|  |  | *Culex spp.* | 45 | 0.0222 | 0 | 0.149 | 0 | 1 |
|  |  | *Culex tritaeniorhynchus* | 45 | 11.5111 | 0 | 32.974 | 0 | 179 |
|  |  | *Culex vishnui* | 45 | 48.8667 | 1 | 190.650 | 0 | 1208 |
|  |  | *Culex whitmorei* | 45 | 0.0222 | 0 | 0.149 | 0 | 1 |
|  |  | *Mansonia annulifera* | 45 | 0.7333 | 0 | 2.220 | 0 | 13 |
|  |  | *Mansonia indiana* | 45 | 0.6667 | 0 | 1.954 | 0 | 11 |
|  |  | *Mansonia spp.* | 45 | 0.0222 | 0 | 0.149 | 0 | 1 |
|  |  | *Mansonia uniformis* | 45 | 0.9111 | 0 | 3.350 | 0 | 20 |
|  |  | *Tripteroides spp.* | 45 | 0.0222 | 0 | 0.149 | 0 | 1 |
|  | rural | *Aedes aegypti* | 45 | 0.0444 | 0 | 0.208 | 0 | 1 |
|  |  | *Aedes albopictus* | 45 | 0.6667 | 0 | 1.796 | 0 | 10 |
|  |  | *Anopheles peditaeniatus* | 45 | 0.0222 | 0 | 0.149 | 0 | 1 |
|  |  | *Culex quinquefasciatus* | 45 | 0.6667 | 0 | 1.206 | 0 | 5 |
|  |  | *Culex spp.* | 45 | 0.0889 | 0 | 0.288 | 0 | 1 |
|  |  | *Culex vishnui* | 45 | 0.3333 | 0 | 0.798 | 0 | 4 |
|  |  | *Mansonia spp.* | 45 | 0.0222 | 0 | 0.149 | 0 | 1 |
|  |  | *Tripteroides spp.* | 45 | 0.0889 | 0 | 0.358 | 0 | 2 |
|  |  | *Aedes poicilius* | 45 | 0.0444 | 0 | 0.208 | 0 | 1 |
|  |  | *Aedes spp.* | 45 | 0.0889 | 0 | 0.358 | 0 | 2 |
|  |  | *Anopheles annularis* | 45 | 0.0222 | 0 | 0.149 | 0 | 1 |
|  |  | *Anopheles culicifacies* | 45 | 0.1333 | 0 | 0.505 | 0 | 3 |
|  |  | *Anopheles dirus* | 45 | 0.0222 | 0 | 0.149 | 0 | 1 |
|  |  | *Anopheles maculatus* | 45 | 0.0889 | 0 | 0.288 | 0 | 1 |
|  |  | *Anopheles minimus* | 45 | 0.1778 | 0 | 0.442 | 0 | 2 |
|  |  | *Anopheles spp.* | 45 | 0.0667 | 0 | 0.330 | 0 | 2 |
|  |  | *Armigeres subalbotus* | 45 | 1.6000 | 0 | 3.313 | 0 | 20 |

*Abbreviations: CDC-LT; CDC light trap, BGP; BG-Pro mosquito trap, IBG; Invert BG-Pro mosquito trap, N; Number of capture day.*
